# Hellebrigenin anti-pancreatic cancer effects based on apoptosis and autophage

**DOI:** 10.7717/peerj.9011

**Published:** 2020-05-08

**Authors:** Xiaolu Wei, Jing He, Bo Gao, Lingyu Han, Yingqiu Mao, Haiyu Zhao, Nan Si, Hongjie Wang, Jian Yang, Baolin Bian

**Affiliations:** 1 Institute of Chinese Materia Medica, China Academy of Chinese Medicine Sciences, Beijing, China; 2 School of Chinese Materia Medica, Beijing University of Chinese Medicine, The Key Unit of Exploring Effective Substances of Classical and Famous Prescription of SATCM, Beijing, China; 3 China Resources Sanjiu Medical and Pharmaceutical Co. Ltd., Shenzhen, China; 4 Beijing University of Chinese Medicine, Beijing, China

**Keywords:** Pancreatic cancer, Hellebrigenin, Antitumor

## Abstract

Hellebrigenin is a natural product found in the toad skin secretions and plants of Urginea, including Hellebores and Kalanchoe genera. It has been shown to be active against Leishmania chagasi promastigotes and Trypanosoma cruzi trypomastigotes and also reported to play an anti-tumor effect on several cancer cell lines in vitro, including pancreatic cancer. This study is aimed to investigate the effects of Hellebrigenin on pancreatic carcinoma cells, SW1990 and BxPC-3 in vitro and its molecular mechanism involved in antitumor activities. Our results showed that Hellebrigenin effectively inhibited the proliferation of SW1990 and BxPC-3 cells in dose- and time-dependent manner. Flow cytometry results showed that Hellebrigenin induced the G0/G1 arrest in both of SW1990 and BxPC-3 cells and promoted cell early apoptosis and autophagy according to morphological observation. Immunofluorescence staining results further confirmed that cell apoptosis and autophagy also increased upon the Hellebrigenin treatment. Moreover, higher dose of Hellebrigenin further increased the cell apoptosis rate while decrease the mitochondrial membrane potential 24 h after treatment. The autophagy rate increased 48 h after treatment with significant difference (*P* < 0.05). Western blot analysis showed that the expression of caspase 3, 7, cleaved caspase 7, Atg 12, LC3 proteins were increased in SW1990 cell after treatment with Hellebrigenin. In addition, increasing expression of caspase 3, 7, 9, PARP, cleaved caspase 3, 7, 9, PARP, the sub basic protein of the PI3K family, Beclin-1, LC 3, Atg 3, 5, 12, 16 L were also observed after BxPC-3 cells treated with Hellebrigenin. In summary, this study reported for the first time that Hellebrigenin effectively induced autophagy and apoptosis especially the early apoptosis in SW1990 and BxPC-3 cells.

## Introduction

Pancreatic cancer ranks the fourth or fifth leading cause of cancer-related death in the developed countries and widely known for its high mortality rate (Maisonneuve, 2016; Hidalgo et al., 2011). In China, it is one of the ten malignant tumors with high fatality rate, and the incidence of pancreatic cancer keeps increasing about six times in the past 20 years (Guo, Cui & Liu, 2013). The clinical manifestations of pancreatic cancer include abdominal distension, indigestion, loss of appetite, weight loss, abdominal pain or back pain, jaundice, fatigue, and individual fever etc. Occult pancreatic cancer is difficult to detect early and it is featured as aggressive, quickly progress, and highly malignant which usually diagnosed at an advanced stage with poor prognosis. Although the detection and treatment developed rapidly in the past few decades, the 5-year survival rate is only about 5% (Hidalgo et al., 2011; Adiseshaiah et al., 2016). Although surgery can be the prospective cure, the resection rate is relatively low due to the asymptomatic of early stage (Maisonneuve, 2016). The fluorouracil chemo-radiation and gemcitabine chemotherapy are regarded as the standard first-line treatments for pancreatic tumors (Stathis & Moore, 2010). However, the benefits were limited due to their intrinsic resistance to chemotherapeutic agents and their toxicity (Hartel et al., 2006; Andersson et al., 2009; Vernerey et al., 2016). Thus, exploring effective treatments to save the lives of patients and improve the life quality are the top priority.

Hellebrigenin is a natural product, which is found in the skin secretions from toads (*Bufo bufo gargarizans and Bufo melanostictus, Rhinella viridis*) and Urginea plants, such as Helleborus and Kalanchoe genera. Hellebrigenin, one of bufadienolides belonging to the cardioactive steroids family, like digoxin and ouabain, has been shown to be active against protozoa (like *Leishmania chagasi promastigotes* and *Trypanosoma cruzi trypomastigotes* (Tempone et al., 2008)) and also showed toxicity to several cancer cell lines in vitro, including colon cancer (HCT-8) (Cunha-Filho et al., 2010), lung cancer (A549) (Liu et al., 2016), leukemia (HL-60) Cunha-Filho et al. (2010) and breast cancer (MCF-7 and MDA-MB-231) (Cunha-Filho et al., 2010). Banuls et al. (2013) reported that the anticancer effect of Hellebrigenin may be related to the inhibition of Na^+^/K^+^-ATPase complexes. Cunha-Filho et al. (2010) showed the cytotoxic effect of hellebrigenin to HL-60 cells without DNA damage or oxidative damage. Wang et al. (2011) reported that Hellebrigenin is a water-soluble chemical component of *B. gargarizans* skin water extract, which has a positive clinical curative effect for advanced digestive tract cancer and hepatitis B. The antitumor activity screened in vitro also indicated that this water extract of toad skin had signifcicant inhibitory effects on A-549 colon cancer cells, and HCT-8 lung cancer cells (Wang et al., 2011). Deng et al. (2014) reported that hellebrigenin is also toxic against the liver cancer cells HepG2 and confirmed that hellebrigenin is a bioactive component of Venenum Bufonis which has anti-hepatoma activity. Meanwhile, hellebrigenin induces DNA damage, triggers cell cycle arrest at G2/M phase and finally triggers cell apoptosis via AKT signaling.

However, the anticancer effect and the involved mechanism in pancreatic cancer cells are still under investigation. This study aimed to evaluate the antitumor effect of Hellebrigenin in human pancreatic carcinoma SW1990 and BxPC-3 cells, and clarify the possible molecular mechanism of Hellebrigenin involved in the toxicity to pancreatic cells.

## Materials and Methods

### Drug and reagents

Hellebrigenin is purchased from Baoji Herbest BioTech Co. Ltd. (Baoji, China). Purities of all compounds were above 96% by HPLC analysis. HPLC grade acetonitrile (Fisher, Fairlawn, NJ, USA) and MS-grade formic acid (ROE Scientific Inc., Newark, DE, USA) were used for UHPLC–ESI–MS/MS analysis.

RPMI1640 maximal medium, DMEM/F12 maximal medium, Penicillin Streptomycin, phosphate-buffered saline (PBS), 0.25% EDTA-trypsin, Fetal bovine serum (FBS), 3-(4,5)-dimethylthiahiazo (-z-y1)-3,5-diphenyte- trazoliumromide (MTT) were purchased from Gibco (Grand Island, NY, USA). Annexin V-FITC/PI apoptosis detection kit was obtained from Becon Dickinson Facsalibur, Franklin Lakes, NJ, USA. RT-PCR kit (Ampliqon, Odense, Denmark), Trizol (Invitrogen, Carlsbad, CA, USA), 5,5′,6,6′-tetrachloro-1,1′,3,3′-tetraethylbenzimidazolcarbocyanine iodide(JC-1), monodansylcadaverine (MDC) and 3-methyladenine (3-MA) were purchased from Sigma–Aldrich (St. Louis, MO, USA).

### Cell line and cell culture

Human pancreatic cancer cell lines, SW1990 and BxPC-3 were obtained from Cell Resource Center, IBMS, CAMS/PUMC (Beijing, China). SW1990 cells were cultured in RPMI 1640 maximal medium (Gibco, Grand Island, NY, USA) while BxPC-3 cells were cultured in DMEM/F 12 maximal medium (Gibco, Grand Island, NY, USA) containing 10% inactivated fetal bovine serum (Gibco, Grand Island, NY, USA) (56 °C, 30 min), penicillin (100 units/mL) and streptomycin (100 units/mL) (Gibco, Grand Island, NY, USA) in a humidified atmosphere with 5% CO_2_ at 37 °C.

### Cell proliferation assay

MTT dye reduction assay (Sigma, St. Louis, MO, USA) was carried out to detect the viability of SW1990 and BxPC-3 Cells as previously reported (Dai et al., 2012). Briefly, cells were seeded into a 96-well plate at a density of 1 × 10^4^ cells/well, cultured for 12 h, then treated with Hellebrigenin with different concentration (0,6, 12, 24, 48, 96 nM in SW1990 and 0, 3.125, 6.25, 12.5, 25, 50 nM in BxPC-3) for 0 to 96 h. At the end of the treatment, 10 μL (50 µg) MTT solution was added into the cells and incubated for another 4 h. 200 μL Dimethylsufloxide (DMSO) was added to each well after removal of the supernatant. After shaking for 10 min, cell viability was measured at the absorbance of 490 nm using an Enzyme-labeling instrument (Multiskan. Go, Thermo Scientific, Ratastie, Finland). The experiments were repeated for three times. Cell growth curve was completed using time as the abscissa and a value (mean ± SEM) as the ordinate.

### Cell cycle analysis

The cell cycle distribution was determined by staining cell with Cycle TESTTM PLUS DNA reagent Kit (BD Biosciences, NJ, USA) and then the cell cycle phases were analyzed by flow cytometer following the manufacturer’s instructions. SW1990 and BxPC-3 cells were treated with Hellebrigenin at various concentrations (24, 48, 96 nM in SW1990 and 7.5, 15, 30 nM in BxPC-3). A total of 48 h after treatment, both floating and attached cells were collected by centrifugation and fixed in 99% cold ethanol for overnight at 4 °C. Then, cells were stained with a fluorochrome solution containing 50 μg/mL PI, 3.4 mM sodium citration, 20 μg/mL RNase A and 1% Triton X-100 in the dark at room temperature for 30 min. Flow cytometry analysis was performed to analyze cell cycle using an EPICS XL flow cytometer (Beckman Coulter, Brea, CA, USA).

### Morphological observation by transmission electron microscope

Uranyl acetate and lead citrate staining were performed to detect the changes in cell morphological. Briefly, adherent SW1990 and BxPC-3 cells were treated with Hellebrigenin (48 nM in SW1990 and 15 nM in BxPC-3). A total of 48 h after treatment, the cells were collected and digested with pancreatin and fixed in ice-cold 4% glutaraldehyde at 4 °C for overnight. To prepare ultra-thin copper sections, the cells were washed twice with PBS, and fixed with 1% osmium acid for 1 h, dehydrated by acetone and embedded in epoxide resin. Then the sections were staining with uranyl acetate and lead citrate. At last, the staining were examined with a Hitachi-800 transmission electron microscope (Hitachi, Tokyo, Japan) (Chen et al., 2016).

### Morphological detection by immunofluorescence

The Hoechst 33258 and MDC are two kinds of immunofluorescence dye which were used to observing the cell apoptosis and autophagy (Zhang, Li & Liu, 2010). Approximately 5 × 10^4^ cells per well were seeded into 24-well plates, cultured for overnight, then treated with different concentration of Hellebrigenin (24, 48, 96 nM in SW1990 and 7.5, 15, 30 nM in BxPC-3), with cells treated with PBS as blank control. After 48 h incubation, the cells were fixed in cold 4% Para-formaldehyde at 4 °C for 15 min, washed with cold PBS twice, centrifuged at 1,000 rpm for 5 min and then supernatant was discarded. The cells were added with Hoechst 33258 (10 µg/mL) and MDC (50 µM) dye separately, and then cells were placed the dark at room temperature for 10 min, washed with PBS twice, centrifuged at 1,000 rpm for 5 min, discarded supernatant and then air dry. The cell staining was observed under the fluorescence microscope (BX-60; Olympus, Tokyo, Japan) and images were taken. The fluorescence intensity was observed under 380 nm for excitation wavelength, 530 nm for emission wavelength.

### Detection of apoptosis and mitochondrial membrane potential

Cell apoptosis was detected by Annexin-V-FITC/PI double staining assay in SW1990 and BxPC-3 cells with Annexin V–FITC/PI apoptosis detection kit (Cell Signaling Technology, Boston, MA, USA) following the manufacturer’s instructions. The cells were seeded into 6-well plates at a density of approximately 5 × 10^5^ cells per well, and cultured overnight. Then, the cells were treated with different concentrations of Hellebrigenin (24, 48, 96 nM in SW1990 and 7.5, 15, 30 nM in BxPC-3), with PBS treatment as blank control. A total of 24 h after incubation, cells were harvested, washed with PBS, and resuspended in Annexin-V binding buffer. The cells was stained with 10 μL of Annexin V-FITC and 10 μL of PI for at least 20 min at room temperature in the dark, and then cell apoptosis were analyzed by Flow Cytometry (Becton Dickinson, SanJose, CA, USA) within 30 min of staining.

Hellebrigenin induced changes in mitochondrial membrane potential were detected by using the fluorescent probe JC-1 (5,5′,6,6′-tetrachloro-1,1′,3,3′-tetraethylbenzimidazolcarbocyanine iodide) as described previously (Ye et al., 2016). The cells were plated into 6-well plates at a density of approximately 5 × 10^5^ cells per well. After cultured overnight, the cells were treated with different concentrations of Hellebrigenin (24, 48, 96 nM in SW1990 and 7.5, 15, 30 nM in BxPC-3). After 24 h treatment, cells were harvested, washed with PBS, and incubated with 5 μM JC-1 in 500 μL of PBS in the dark at 37 °C for 30 min. After washed with PBS three times, cells were analyzed immediately by the Flow Cytometry.

### Detection of autophagy

Cyto ID^®^ staining tests assay was performed to detect the autophagy of SW1990 and BxPC-3 cells by using Cyto ID^®^ Autophagy detection kit (Enzo Life Sciences, Inc., Farmingdale, NY, USA) following the manufacturer’s instructions. The cells were seeded into 100 mm Petri dish at a density of 5 × 10^5^ cells per dish. After cultured for overnight, the cells were treated with different concentrations of Hellebrigenin (24, 48, 96 nM in SW1990 and 7.5, 15, 30 nM in BxPC-3). After 24 h incubation, cells were harvested, washed with PBS, resuspended in 1× assay buffer and stained with Cyto-ID^®^ green dye for 30 min at room temperature in the dark. Then, the cells were centrifuged at 1,000 rpm for 5 min, and then supernatant was discarded. The cells were washed with 1× assay buffer, and centrifuged at 1,000 rpm for 5 min. The cells were resuspended in 1× assay buffer, and analyzed by Flow Cytometry (Becton Dickinson, SanJose, CA, USA) within 30 min of staining.

### Western blot analysis

SW1990 and BxPC-3 cells (1 × 10^7^ cells) were treated with various concentrations of Hellebrigenin (24, 48, 96 nM in SW1990 and 7.5, 15, 30 nM in BxPC-3) for 48 h. Then western blot was performed to check the apoptotic protein changes. While SW1990 and BxPC-3 cells (1 × 10^7^ cells) were treated with high concentration of Hellebrigenin for different times (0, 6, 12, 18, 24 and 48 h). After indicated treatments, cells were collected and cell lysates were prepared using RIPA buffer (50 mM Tris-HCl pH 7.4, 150 mM NaCl, 1% NP-40, 0.1% SDS, 1 mM PMSF). Protein concentrations were determined and equal amounts of total proteins from each sample were separated on SDS–PAGE and transferred to Polyvinylidene Fluoride (PVDF) membranes at 180 mA for 1 h. Membranes were blocked with 5% fat-free milk in TBST (10 mM Tris-HCl, pH 7.4, 150 Mm NaCl, 0.05% Tween-20) for 1 h and then incubated with the related primary antibodies for overnight. After washed with TBST for four times, 5 min each time, the membranes were incubated with corresponding HRP-conjugated secondary antibodies. GAPDH was used as an internal control. Protein expressions were detected by chemiluminescence with the enhanced chemiluminescent detection kit following the manufacturer’s protocols.

### Statistical analysis

All data were expressed by mean ± S.E.M. SPSS 13.0 for Windows was used to analyze the statistical analyses. Student’s *t*-test was used to compared the difference between two groups while, one-way analysis of variance (ANOVA) was used to analyze statistical differences between more than two groups. *P* <0.05 was recognized as statistically significant.

## Results

### Hellebrigenin inhibits pancreatic cancer cell proliferation

**Figure 1:**
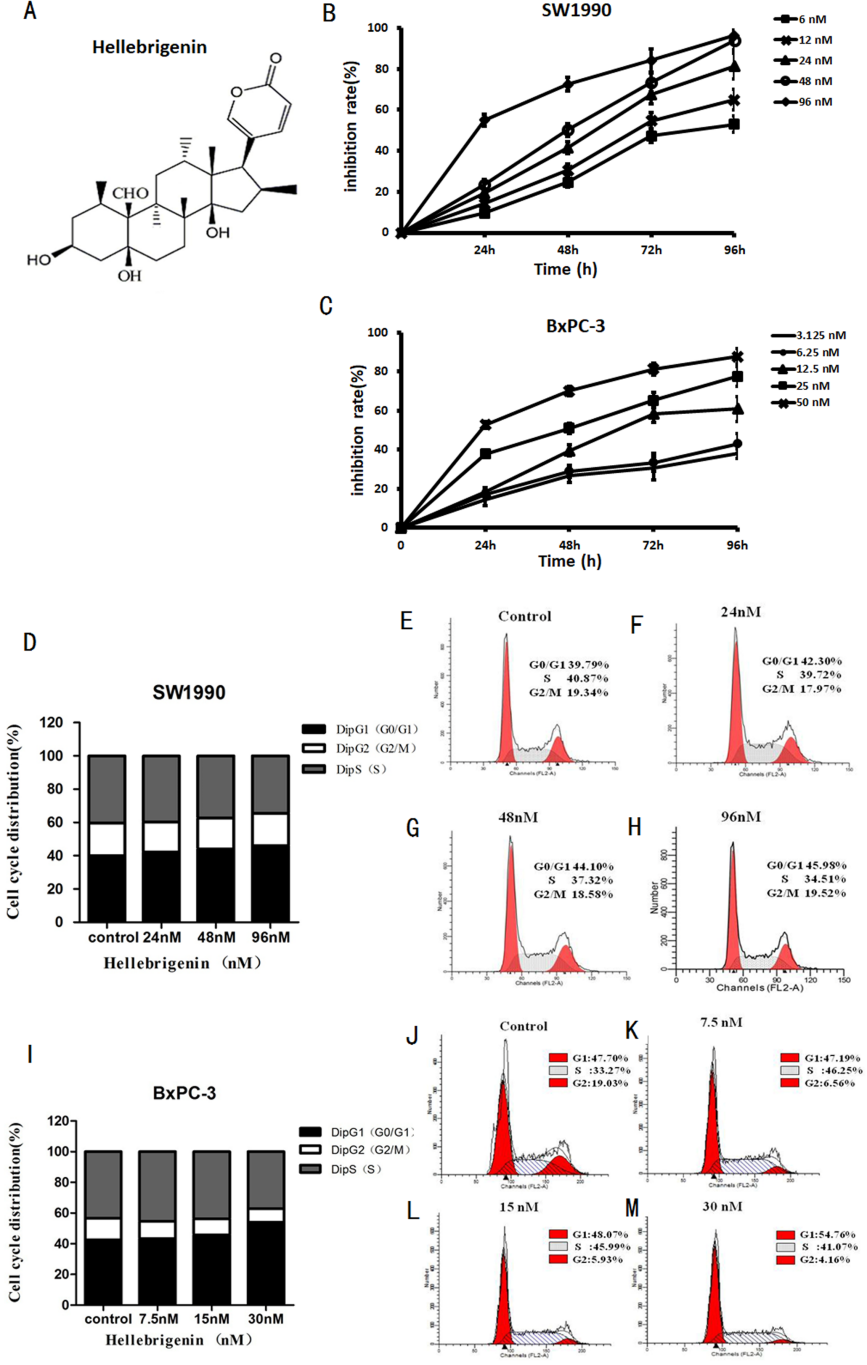
The Hellebrigenin chemical formula and the anticancer effect. (A) The chemical formula of Hellebrigenin. (B and C) The Growth inhibiting effects of Hellebrigenin on SW1990 and BxPC-3 cells. SW1990 and BxPC-3 cells were treated with different concentrations for 0–96 h. Cell viability was determined by MTT method. This assay was performed in triplicate. Dose- and time-dependent inhibition of cell growth could be observed after 96 h (*P* < 0.05, ANOVA analysis). (D–H) SW1990 Cells were treated with 24, 48 and 96 nM of Hellebrigenin for 48 h. The cell cycle distribution was determined using flow cytometry analysis and cell cycle distribution was quantified. Data were presented as the mean ± SEM of three independent experiments. *P < 0.05, significantly different compared with control treatment. (I–M) BxPC-3 Cells were treated with 24, 48 and 96 nM of Hellebrigenin for 48 h. The cell cycle distribution was determined using flow cytometry analysis and cell cycle distribution was quantified. Data were presented as the mean ± SEM of three independent experiments. *P < 0.05, significantly different compared with control treatment.

The pancreatic cancer cells SW1990 and BxPC-3 were treated with different concentrations of Hellebrigenin for 0 to 96 h. Then, cell viability was determined by MTT assay to examine the anti-proliferative effect of Hellebrigenin (Fig. 1A). As shown in Fig. 1B, the Hellebrigenin inhibited the SW1990 and BxPC-3 cells’ viability/proliferation in a dose- and time-dependent manner according to MTT assay. The inhibition rate of Hellebrigenin on SW1990 and BxPC-3 cell growth was as high as (96.45 ± 2.38)% and (87.71 ± 3.45)% respectively, when the cells were treated for 96 h with highest dose of Hellebrigenin.

Then, we examined the effect of Hellebrigenin on cell cycle distribution after the cells exposed to various concentrations of Hellebrigenin for 48 h. The results showed that the higher dose of Hellebrigenin increased the G0 period cell number, while blocked the S period cell number, leading to the G0/G1 phase cell accumulation after pancreatic cancer cells were treated with Hellebrigenin (Figs. 1C and 1D). Thus, these results suggested that Hellebrigenin effectively caused G0/G1 arrest and may induce cell early death.

### Morphological observation of apoptosis and autophagy on cells induced by Hellebrigenin

**Figure 2:**
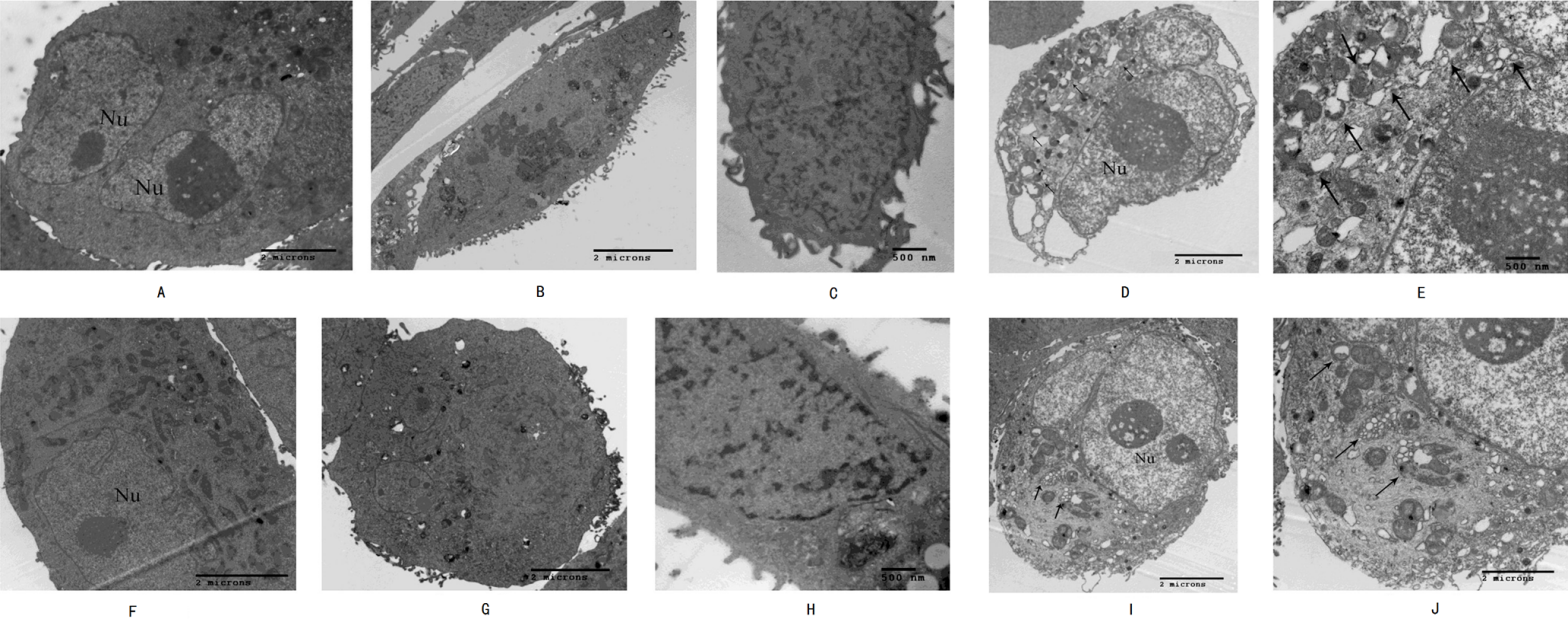
Morphological observation of SW1990 cells by transmission electron microscopy after treatment with Hellebrigenin. (A) Normal SW1990 cells (8,000×); (B) Karyopyknosis and chromatic agglutination (15,000×); (C) Formation of apoptotic body (6,000×); (D) Characteristic ultrastructural morphology of autophagy in SW1990 cells (6,000×); (E) Autophagic vacuoles in SW1990 cells (20,000×); (F) Normal BxPC-3 cells (12,000×); (G) Karyopyknosis and chromatic agglutination (8,000×); (H) Formation of apoptotic body (25,000×); (I) Characteristic ultrastructural morphology of autophagy in BxPC-3 cells (6,000×); (J) Autophagic vacuoles in BxPC-3 cells (12,000×).

High-resolution transmission electron microscopy was used to observe the cell morphology and the results showed that normal pancreatic cancer cells SW1990 and BxPC-3 had a round and regular in shape with chromatin margination in few tumor cells (Figs. 2A and 2F). After the cells treated with Hellebrigenin for 48 h, the nuclei showed chromatin condensation, and were accumulated at the inner edge of nuclear membrane (Figs. 2B and 2G). The typical morphologies of apoptotic SW1990 and BxPC-3 cells such as chromatic agglutination and nuclear fragmentation, mitochondrial swelling, and apoptotic bodies formation, could be observed in the Hellebrigenin treated group (Figs. 2C and 2H). In addition, autophagy was also detected in the Hellebrigenin treatment group, indicating by characteristic ultra-structural morphology. Abundant autophagic vacuoles are trapped in cytoplasm and organelles, including mitochondria and endoplasmic reticulum (Figs. 2D, 2E and 2I, 2J). Altogether, these results showed that both autophagy and apoptosis were activated in SW1990 and BxPC-3 cells after Hellebrigenin treatment.

### Morphological detection of apoptosis and autophagy in cells by immunofluorescence

**Figure 3:**
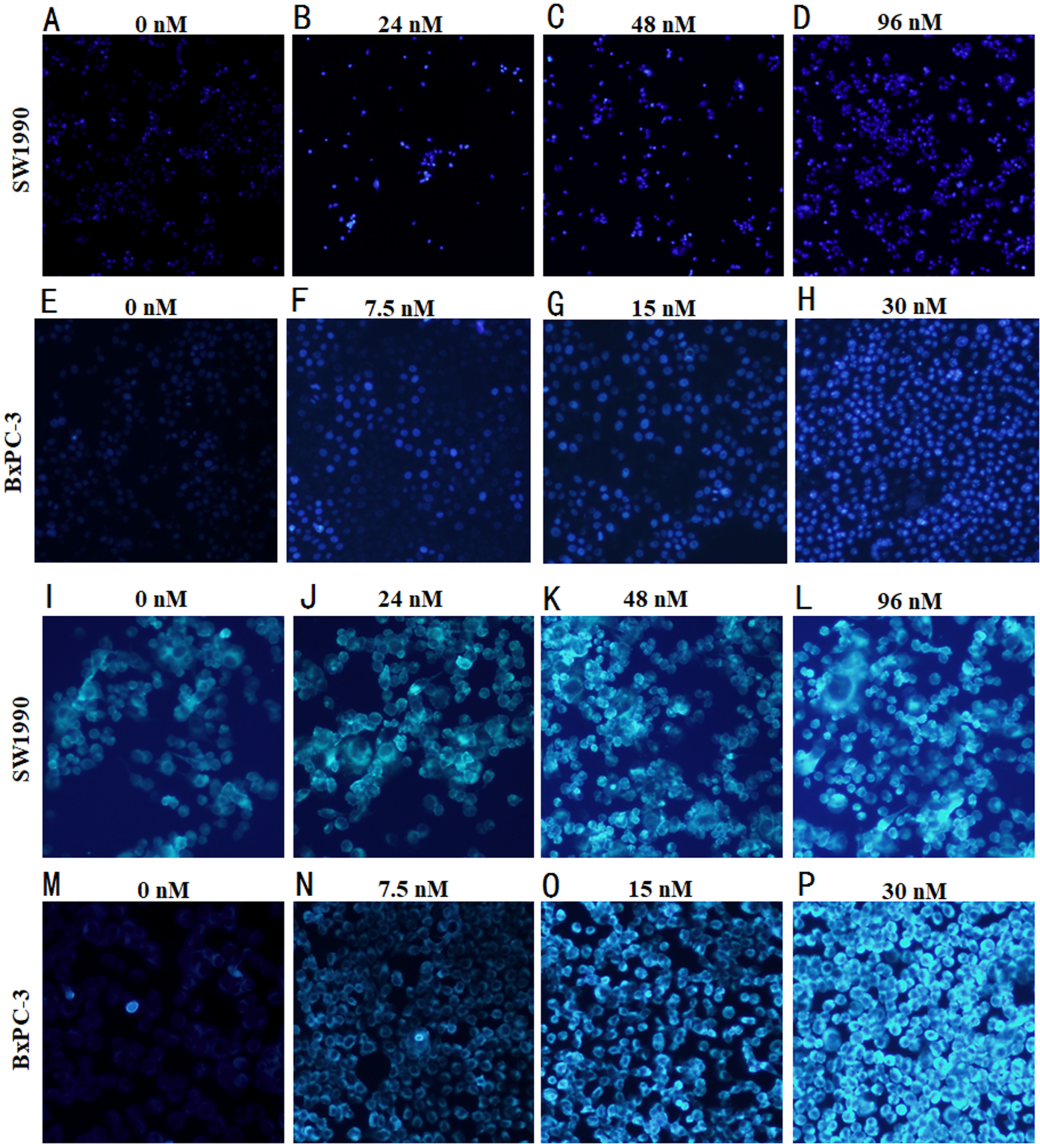
Morphological detection of apoptosis and autophagy in cells by immunofluorescence. (A–H) The Hoechst 33258 Staining morphological changes of apoptosis in SW1990 and BxPC-3 cells by fluorescence microscope after treatment with different concentrations of Hellebrigenin. The cells were labeled with 10 µM Hoechst 33258 in phosphate buffered saline (PBS) at room temperature for 10 min. (I–P) MDC-labeled autophagic vacuole in SW1990 and BxPC-3 cells by fluorescence microscope after treatment with different concentration of Hellebrigenin. Autophagic vacuoles were labeled with 50 µM MDC in PBS at room temperature for 10 min.

Hoechst 33258, when binds to dsDNA emits blue flurorescence, was used for the nuclear counter staining to detect the cell apoptosis under fluorescence microscope. After the pancreatic cancer cells treated with Hellebrigenin for 48 h, the apoptosis of SW1990 and BxPC-3 cells was observed by morphological changes through Hoechst33258 staining. The results showed that the nuclear of blank control group cells showed normal blue fluorescence, while the nuclear of the Hellebrigenin treated group cells showed the increased aggregation of chromatin increased and brighter blue fluorescence in a Hellebrigenin dose dependent manner (Fig. 3A).

Monodansylcadaverine (MDC) staining is an autophagic vesicle tracer that specifically binds to Atg8 on the autophagic vesicle membrane, the positive result can represent autophagic vesicles. The number of autophagic vesicles can be observed under a fluorescence microscope (Biederbick, Kern & Elsässer, 1995). After the pancreatic cancer cells treated with Hellebrigenin for 48 h, the autophagy in SW1990 and BxPC-3 cells observed by MDC staining indicated that the blank control group showed normal dark green fluorescence, while the Hellebrigenin treated cells showed an increased green fluorescence, indicating that the number of autophagic vesicles increased in the cell in a Hellebrigenin dose dependent manner (Fig. 3B).

### Flow cytometry analysis of cell apoptosis induced by Hellebrigenin

**Figure 4:**
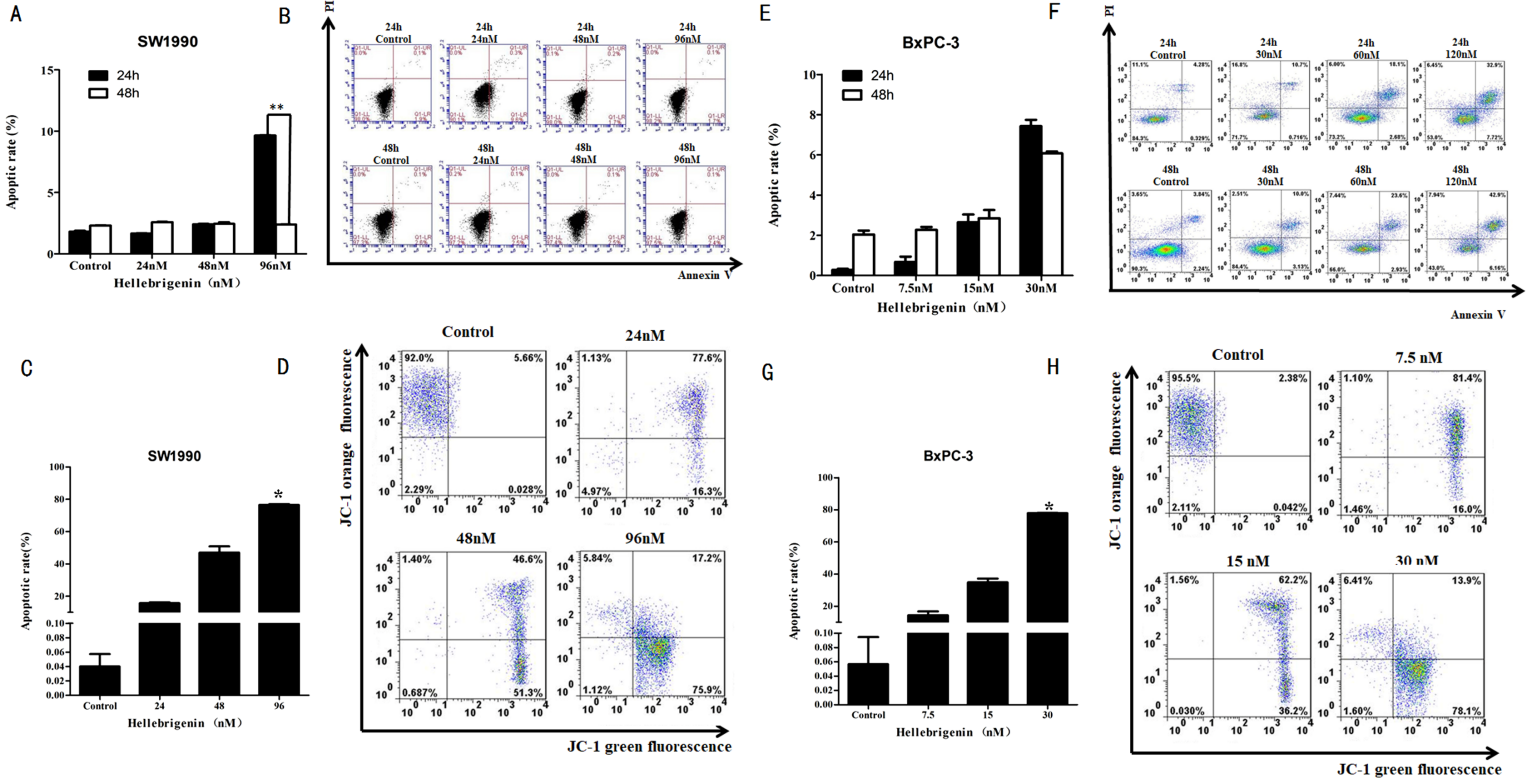
The flow cytometry analysis for SW1990 and BxPC-3 cells. (A and B) After various Hellebrigenin group (24, 48, 96 nM) treatment on SW1990 cells at 24 h or 48 h by Annexin V-FITC and PI staining for apoptosis and the column data statistics, *P < 0.05 versus control group. (C and D) After various Hellebrigenin group (24, 48 and 96 nM) treatment on cells at 24 h by JC-1 staining for mitochondrial membrane potential and the column data statistics, *P < 0.05, **P < 0.01 versus control group. (E and F) After various Hellebrigenin group (7.5, 15 and 30 nM) treatment on BxPC-3 cells at 24 h or 48 h by Annexin V-FITC and PI staining for apoptosis and the column data statistics, *P < 0.05 versus control group. (G and H) After various Hellebrigenin group (7.5, 15 and 30 nM) treatment on BxPC-3 cells at 24 h by JC-1 staining for mitochondrial membrane potential and the column data statistics, *P < 0.05, **P < 0.01 versus control group.

First, we used different doses of Hellebrigenin to treat SW1990 and BxPC-3 cells for 24 h and 48 h. Then the cells were stained with Annexin V-FITC/PI combination and detected by flow cytometry (FCM). The cell apoptosis rates were calculated. The results showed that compared with blank control group, as the concentration of Hellebrigenin increased, the cell apoptosis rate was significantly increased when the SW1990 cells were treated for 24 h. The apoptosis rate induced by higher dose group (96 nM) was 9.65%, which is significantly higher than the blank group (*P* < 0.05), while there was no significant change in apoptotic rate when the SW1990 cells were treated for 48 h (Figs. 4A–4B). When the BxPC-3 cells were treated with Hellebrigenin for 24 h and 48 h, the apoptosis rate was profoundly increased in a dose dependent manner compared with the blank control group, and the higher dose treatment (30 nM) for 24 h and 48 h induced the apoptosis rates to 7.43% and 6.08% respectively, which are significantly higher than the blank control (*P* < 0.05) (Figs. 4E–4F).

Next, we detect the apoptosis in SW1990 and BxPC-3 cells treated with different concentrations of Hellebrigenin treated for 24 h, by using the fluorescent probe JC-1 which can bind to the mitochondrial membrane potential (Δψ) and can be checked by FCM. The results demonstrated that the apoptosis rate was higher in Hellebrigenin treated cells in a dose dependent manner. The higher dose with 96 nM treatment of SW1990 and 30 nM treatment of BxPC-3 induced the apoptosis rate to 76.34% and 77.87% respectively, which is significantly higher than the blank control (*P* < 0.05) (Figs. 4C–4D and 4G–4H).

### Flow cytometry analysis of cell autophagy induced by Hellebrigenin

**Figure 5:**
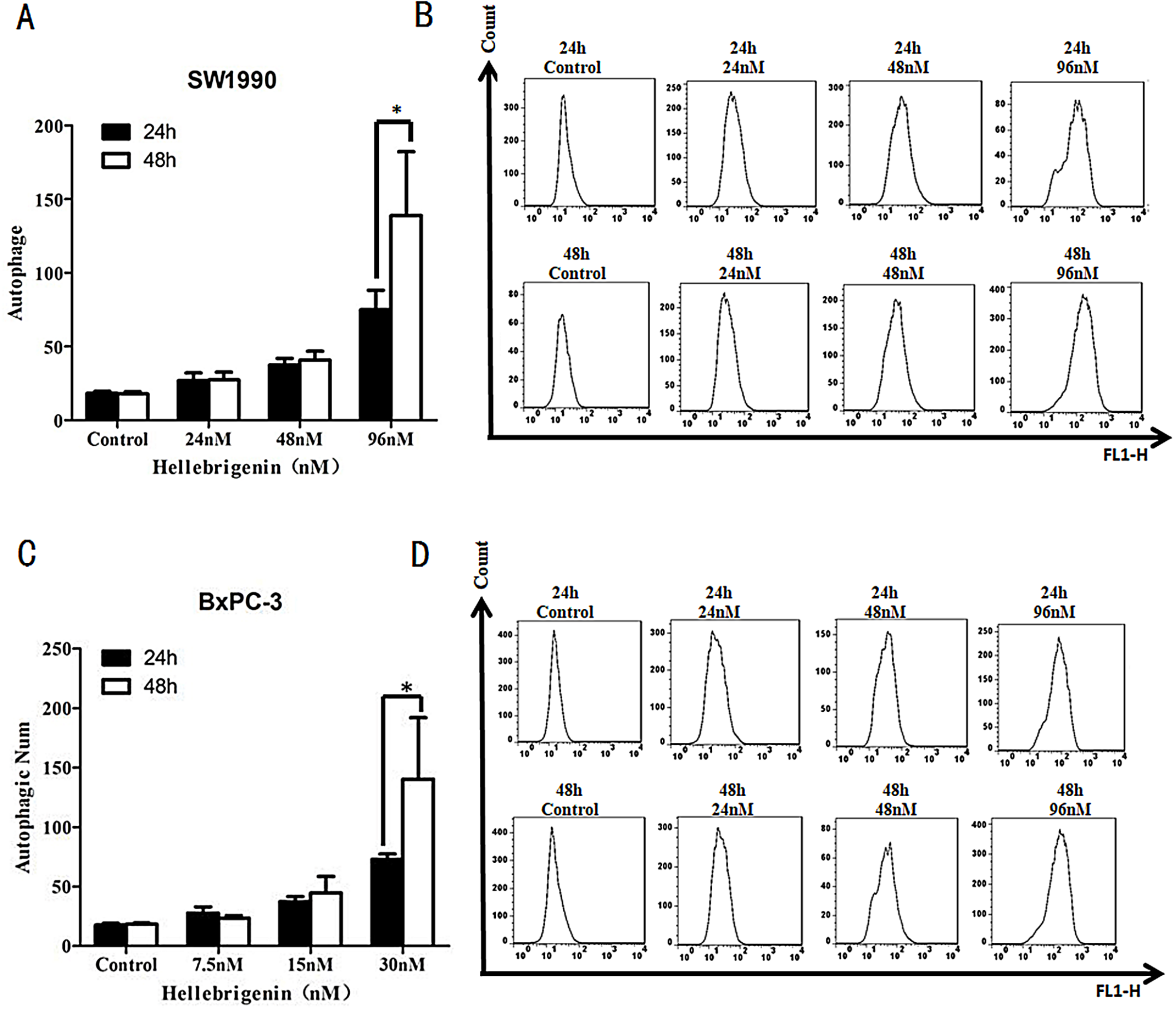
The flow cytometry analysis for SW1990 and BxPC-3 cells. (A and B) After various Hellebrigenin group (24, 48 and 96 nM) treatment on SW1990 cells at 24 h or 48 h by Cyto ID^®^ staining for autophagy states, *P < 0.05 versus control group. (C and D) After various Hellebrigenin group (7.5, 15 and 30 nM) treatment on BxPC-3 cells at 24 h or 48 h by Cyto ID^®^ staining for autophagy states, *P < 0.05 versus control group.

SW1990 and BxPC-3 cells were treated with different doses of Hellebrigenin for 24 h and 48 h, and then the cells were stained with Cyto ID^®^ staining and the autophagy states were detected by FCM to calculate the autophagy rates. The results showed that with the dose increase, Hellebrigenin remarkedly promoted the autophagy in SW1990 and BxPC-3 cells in different degrees and the autophagy rate was significantly increased after the cells were treated for 24 h and 48 h. The autophagy rate of higher concentration group (96 nM in SW1990 and 30 nM in BxPC-3) were 138.9% for SW1990 and 140.3% for BxPC-3 at 48 h, while 74.9% for SW1990 and 72.9% for BxPC-3 at 24 h. Even after treated with Hellebrigenin for 24 h, the rates of autophagy in SW1990 and BxPC-3 cells was significantly higher than that of control group (*P* < 0.05), indicating that Hellebrigenin induces cell autophagy with the time and dose-effect relationship (Figs. 5A and 5B).

### The effects of Hellebrigenin on apoptosis-related proteins

**Figure 6:**
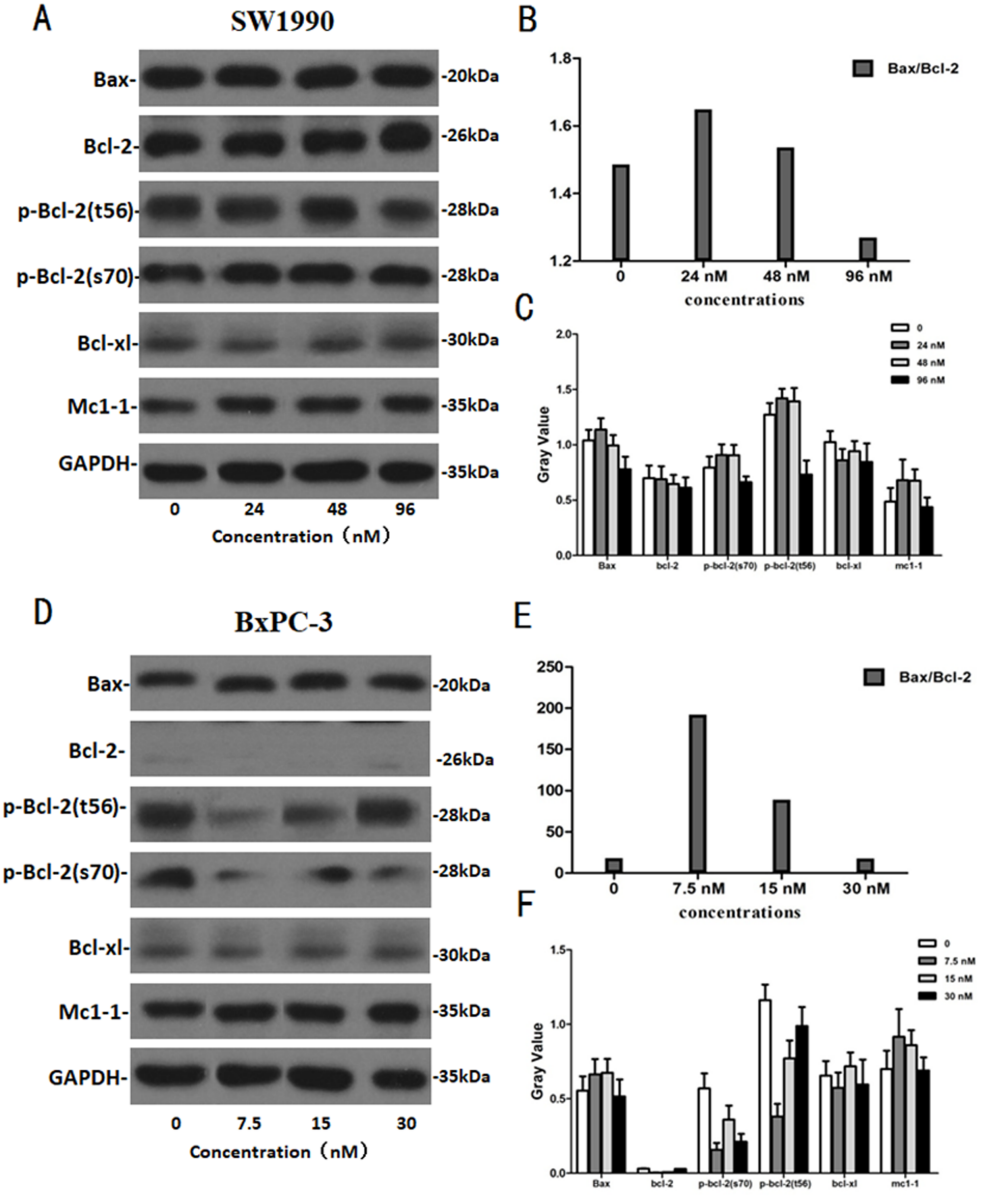
The western blot results of Bax and Bcl-2 protein family for SW1990 and BxPC-3 cells. (A–C) The protein running gel and gray value of Bax and Bcl-2 protein family in SW1990 cells, the ratio of Bax/Bcl-2 indicate that the Hellebrigenin can promote the apoptosis. (D–F) The protein running gel and gray value of Bax and Bcl-2 protein family in BxPC-3 cells, the ratio of Bax/Bcl-2 indicate that the Hellebrigenin can promote the apoptosis.

**Figure 7:**
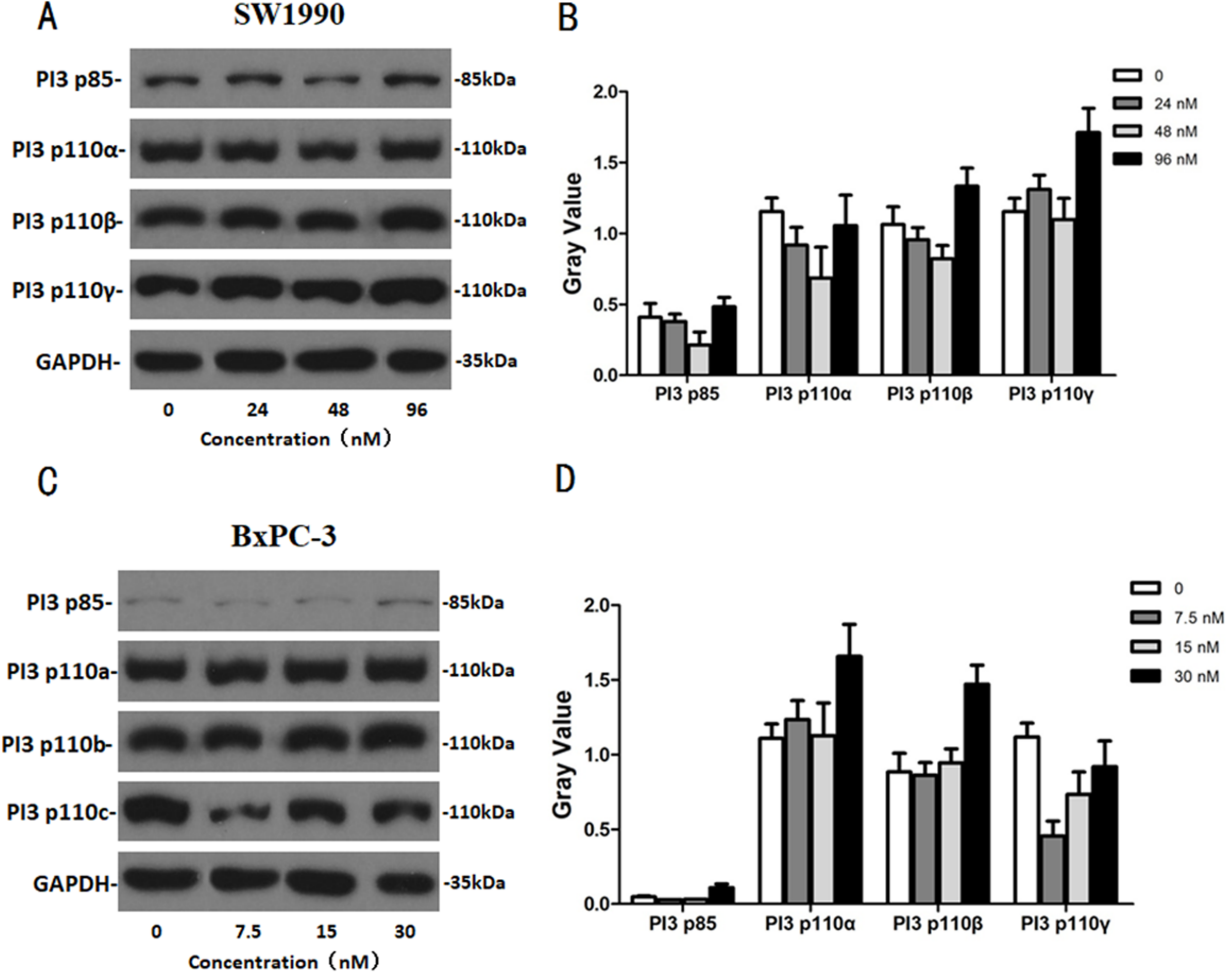
The western blot results of PI3K protein family for SW1990 and BxPC-3 cells. (A and B) The protein running gel and gray value of PI3K protein family in SW1990 cells. (C and D) The protein running gel and gray value of PI3K protein family in BxPC-3 cells.

**Figure 8:**
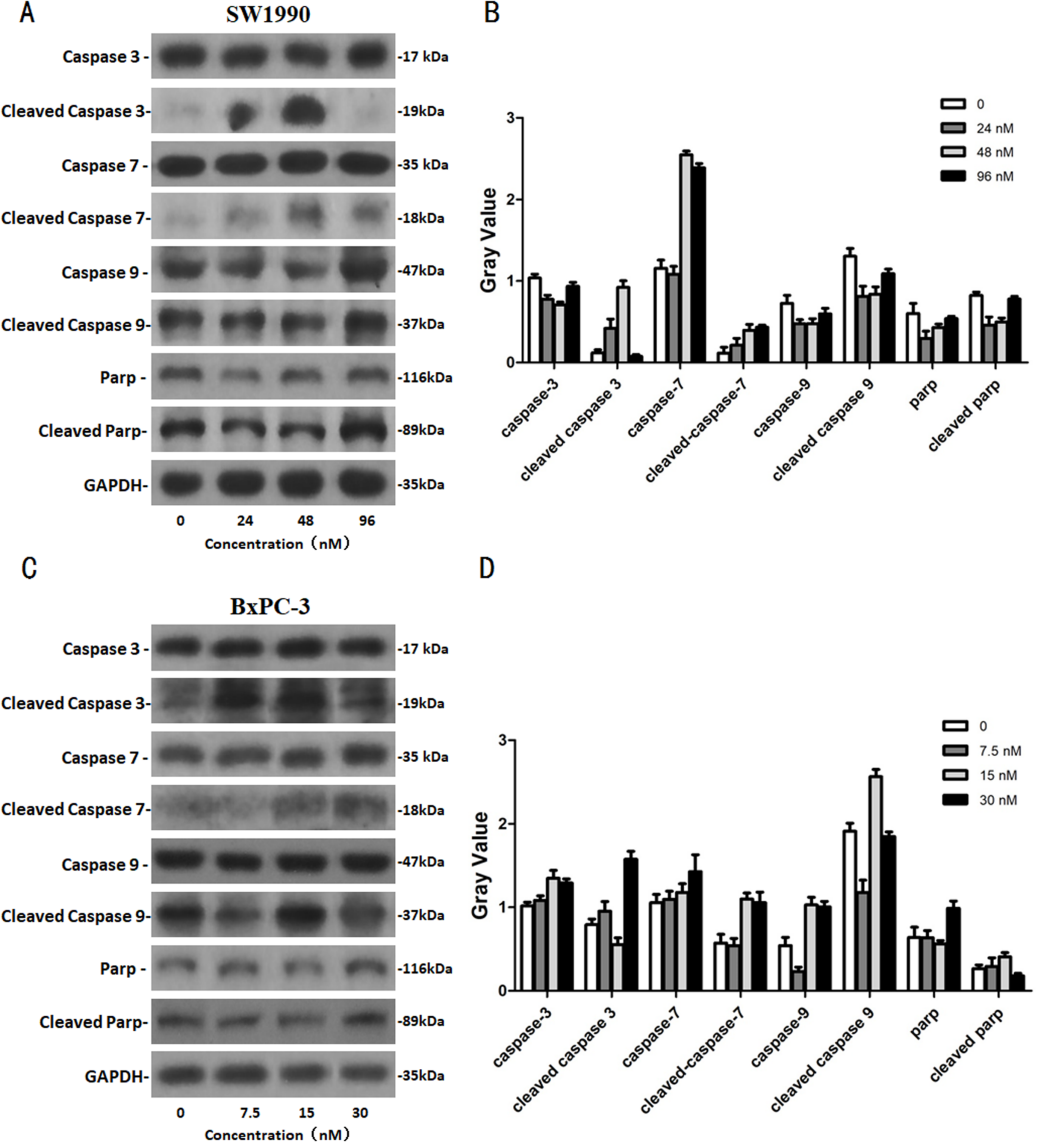
The western blot results of Caspase protein family for SW1990 and BxPC-3 cells. (A and B) The protein running gel and gray value of Caspase protein family in SW1990 cells. (C and D) The protein running gel and gray value of Caspase protein family in BxPC-3 cells.

After SW1990 and BxPC-3 cells treated with different doses of Hellebrigenin for 48 h, Western blot assay was performed to examine the expression of apoptosis-related proteins. The results showed that Hellebrigenin treatment markedly increased the expression of Bax, a pro-apoptotic protein of the Bcl-2 protein family in SW1990 and BxPC-3 cells, decreased the expression of other proteins including Bax, Bcl-xl, and Mc1-1 in Bcl-2 family, and increased the ratio of Bax/Bcl-2 (Fig. 6). The expression of Caspase family and PI3K family proteins in SW1990 and BxPC-3 cells were different. In addition, we also detect other apoptosis-related proteins and the results indicated that the expression of caspase 3, cleaved caspase 3, cleaved caspase 7 and p85, p110β, p110γ in SW1990 cells are increased, while caspase 3, caspase 7, caspase 9, PARP, cleaved caspase 3, cleaved caspase 7, cleaved caspase 9, cleaved caspase PARP and p85, p110α, p110β in BxPC-3 cells are increased (Figs. 7 and 8). Thus, these results further confirmed that Hellebrigenin treatment promoted the pancreatic tumor cells apoptosis via upregulation of apoptosis-related proteins expression.

### The effects of Hellebrigenin on autophagy related proteins

**Figure 9:**
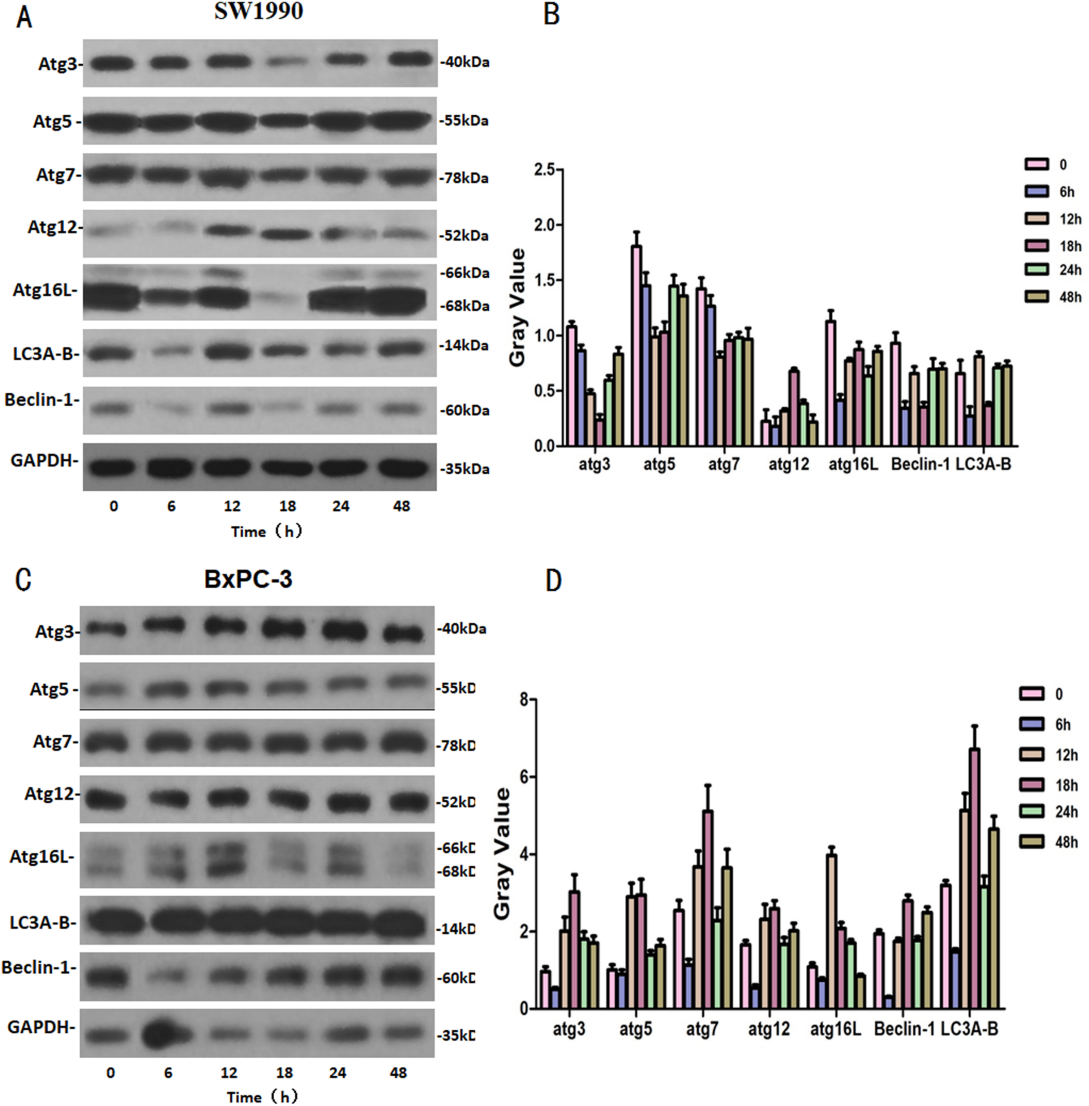
The western blot results of Autophagy protein family for SW1990 and BxPC-3 cells. (A and B) The protein running gel and gray value of Autophagy protein family in SW1990 cells. (C and D) The protein running gel and gray value of autophagy protein family in BxPC-3 cells.

SW1990 and BxPC-3 cells were treated with higher dose of Hellebrigenin for different times. Then, western blot was performed to detect the expression of autophagy-related family proteins. The results showed that the expression of Atg3/5/7/16L and Beclin-1 are decrease while the expression of Atg 12 and LC 3 in SW1990 cells are increased at all the time point after treatment. While, in BxPC-3 cell, the expression of Atg 3, Atg 5, Atg 7, Atg 12, Atg 16L, Beclin-1 and LC3 are all increased in anytime (Fig. 9). Altogether, these results suggested that Hellebrigenin treatment promoted autophagy through upregulation of autophagy related proteins.

## Discussion

The traditional Chinese medicine has the characteristics of multiple components, effects and targets, little toxic and side effects, strong overall regulation and synergistic effect, and has its unique advantages in the treatment of pancreatic cancer (Yang et al., 2015). Hellebrigenin, as a single compound of Huachansu injection, was reported to be active against Leishmania chagasipromastigotes and Trypanosoma cruzi trypomastigotes (Tempone et al., 2008) and also shows toxicity against several cancer cell lines in vitro, which include Colon cancer (HL-60), lung cancer (HCT-8), leukemia (A549), breast cancer (MCF-7), pancreatic cancer (PC-3), oral epidermoid cancer KB cells and HeLa cells (Cunha-Filho et al., 2010; Banuls et al., 2013; Kamano et al., 1998). In our current research, Hellebrigenin inhibited the proliferation of SW1990 cells in a dose- and time-dependent manner. Furthermore, FCM assay suggested that Hellebrigenin effectively caused cell G0/G1 phase arrest and then led to the early cell death.

Apoptosis is a form of programed cell death and is also considered to be the predominant one. The main features of the apoptosis in cells include nuclear fragmentation, chromatin condensation, chromosomal DNA fragmentation and cell shrinkage et al. Later, more and more studies indicated the role of autophagy under normal and pathological conditions, which demonstrated that autophagic cell death is an alternative form of cell death, leading to the reclassification of programed cell death into two types, apoptotic cell death and autophagic cell death (Baehrecke, 2002; Loos et al., 2013). Autophagy is an evolutionarily conserved process that sequesters organelles and long-lived proteins in a double-membrane vesicle (autophagosomes), for subsequent lysosomal degradation (Thorburn & Morgan, 2013). In normal cells, autophagy contributes to the conversion of long-lived proteins and elimination of damaged or aged organelles, thereby maintaining cell homeostasis (Yang et al., 2008; Morselli et al., 2009). Under pathological conditions, autophagy is generally thought to have a prosurvival effect. Recently, there are different evidences showed that autophagy is closely related to tumors and plays an critical role in suppression of human tumor progression (Xiao et al., 1999; Yue et al., 2003). In this study, our morphological observation through the high-resolution transmission electron microscopy showed that, the typical morphologies of both apoptosis and autophagy were shown after the SW1990 cells treated with Hellebrigenin. Apoptosis and autophagy were observed upon combination of a higher fluorescent density and MDC-labeled particles in SW1990 cells after the cells treated with Hellebrigenin for 24 h, indicating the cell death upon treatment. Moreover, flow cytometry data indicated the increased apoptosis rate in pancreatic tumor cells upon treatment with Hellebrigenin. At the same time, the breakdown of mitochondrial membrane potential was also supported that autophagy was activated under the treatment with Hellebrigenin in pancreatic cells, which further confirmed the above phenomenon indicating that Hellebrigenin treatment led to the early apoptosis of pancreatic tumor cells.

We further detected the expression of apoptosis and autophagy-related proteins with western blot. It is well-known that the Bax and Bcl-2 proteins which belongs to Bcl-2 proteins family, play important roles in the activation of the mitochondrial pathway related apoptosis. The Caspase proteins family, belongs to the cysteine acid proteases family and plays a pivotal role on the apoptosis regulation (Tomek, Akiyama & Dass, 2012). The PI3K protein family is involved in the cell functions regulation, such as cell proliferation, differentiation, apoptosis and glucose transportation (Mao et al., 2016; Fujimoto et al., 2009). The LC3 protein serves as a marker protein of autophagosomes (Yoshioka et al., 2008). Beclin-1 which is a homolog of yeast Atg6/Vps30 autophagy related gene, is also involved in autophagy and tumor genesis as well as is an essential factor for autophagosome formation (Kung et al., 2011; Mei et al., 2016). After using western blot to detect the expression of the proteins, including Bcl-2, Caspase and PI3K families and some autophagy indicator proteins, we found that the apoptosis and autophagy in SW1990 and BxPC-3 cells were induced by Hellebrigenin in different ways. In SW1990 cells, Hellebrigenin induced cells apoptosis by activating the expression of caspase 3, 7 and cleaved caspase 7 proteins in the caspase family proteins, activating the PI3K protein of PI3K/Akt/mTOR signaling pathway and increasing the ratio of Bax/Bcl-2, and promoted cells autophagy by activating the expression of Atg12 and LC3. While in BxPC-3 cells, Hellebrigenin induced cells apoptosis with multi-pathway by activating the caspase family of promoter factors capsase9 and cleaved caspase9, the execution factors caspase 3, 7 and cleaved caspase 3, 7, PARP and cleaved PARP protein and increasing the ratio of Bax/Bcl-2, activating the PI3K protein in PI3K/Akt/mTOR signaling pathway, and promoted the cells autophagy by activating the autophagy marker protein in autophagy family and most of the relevant proteins. The autophagy rate of BxPC-3 cells was higher than that of SW1990 cells, which is same as the result of detecting the autophagy rate by flow cytometry.

## Conclusion

MTT assays showed that Hellebrigenin inhibited the proliferation of SW1990 and BxPC-3 cells in vitro in a time- and dose-dependent manner. Both autophagy and apoptosis were activated after the pancreatic cancer cells treated with Hellebrigenin. The morphological observation and immunofluorescence staining showed that the Hellebrigenin treatment induced the G0/G1 phase arrested and the cell early death which is associated with apoptosis and autophagy in pancreatic tumor cells. Annexin-V-FITC/PI double staining and JC-1 staining assays revealed the higher dose of Hellebrigenin treatment increased the cell apoptosis rate at 24 h and decreased the mitochondrial membrane potential, While the Cyto ID^®^ staining tests showed the autophagy rate was increased at 48 h with significant difference (*P* < 0.05). The western blot analysis confirmed the Hellebrigenin inhibited pancreatic cancer cells proliferation by inducing cell apoptosis and activation of autophagy via upreguation of apoptosis related proteins and the autophagic key proteins. However, the way of apoptosis and autophagy in SW1990 and BxPC-3 cells are different, which is needed to be further investigated.

## Supplemental Information

10.7717/peerj.9011/supp-1 Supplemental Information 1 Raw data.

## Additional Information and Declarations

### Data Availability

The following information was supplied regarding data availability: The blots and data are available at figshare: bian (2020): Hellebrigenin data. figshare. Dataset. DOI 10.6084/m9.figshare.11918400.v1.
